# LPS-Induced Inflammation and Preconditioning in Rainbow Trout: Markers of Innate Immunity and Oxidative Stress

**DOI:** 10.3390/ani15243589

**Published:** 2025-12-14

**Authors:** Irina V. Sukhovskaya, Albina A. Tsekova, Nadezhda P. Kantserova, Olga V. Balan, Tamara Y. Kuchko, Svetlana V. Matrosova, Alexander N. Belyaev, Liudmila A. Lysenko

**Affiliations:** 1 Karelian Research Centre of the Russian Academy of Sciences, Institute of Biology, 185910 Petrozavodsk, Russia; kochnevaalbina@gmail.com (A.A.T.); nkantserova@yandex.ru (N.P.K.); ovbalan@mail.ru (O.V.B.); l-lysenko@yandex.ru (L.A.L.); 2 Institute of Biology, Ecology and Agricultural Technologies of the Petrozavodsk State University (PetrSU), 185640 Petrozavodsk, Russia; t_kuchko70@mail.ru (T.Y.K.); matrosovasv@yandex.ru (S.V.M.); kuchko@petrsu.ru (A.N.B.)

**Keywords:** *Oncorhynchus mykiss*, LPS, inflammation, cytokine gene expression, CRP, serum bactericidal activity, antioxidant enzymes

## Abstract

The lipopolysaccharide (LPS)-induced inflammation model is widely used in mammalian studies but remains poorly investigated in a variety of fish species. We reproduce this model in rainbow trout, *Oncorhynchus mykiss*, a species of high economic value, to validate specific inflammatory biomarkers for reliably assessing the health and immune status of farmed fish. The inflammatory process, together with the effect of preconditioning, was modeled using two consecutive intraperitoneal injections of 300 and 600 µg LPS. We assessed innate immunity biomarkers, including the expression of inflammation-related genes (*il1ß*, *il8*), peripheral blood leukocyte profiles, serum bactericidal activity (SBA), and various serum and hepatic biochemical parameters. These parameters comprised the concentration of C-reactive protein (CRP, an acute phase protein), and the activities of antioxidant enzymes (superoxide dismutase, catalase, glutathione-S-transferase) measured at 24 and 96 h post-injection. In trout, LPS-induced effects involved the overexpression of pro-inflammatory interleukins (*il1ß*, *il8*), a left shift in white blood cells (characterized by a prevalence of immature neutrophils), and enhanced SBA. In contrast to warm-blooded animals, LPS challenge in trout did not appear to significantly elevate CRP levels or antioxidant enzyme activity. Further investigations in other fish species are needed to determine whether these are traits specific to trout or common to bony fish. Our findings provide a foundation for developing a biomarker panel suitable for the routine assessment of welfare, early detection of infection-associated inflammation in cultured fish, and the screening of the anti-inflammatory and immunostimulant activities of drugs and feed additives.

## Simple Summary

Many infectious diseases are associated with inflammation. To study inflammation in fish, a validated experimental model is required. We used a lipopolysaccharide (LPS)-induced inflammation model to reproduce inflammation in rainbow trout and evaluate traditional inflammatory biomarkers, including pro-inflammatory cytokines, acute phase proteins, blood bactericidal activity, leukocyte profiles, and oxidative stress-associated enzymes. Interestingly, not all parameters responded to LPS as predicted; notably, C-reactive protein (CRP) and antioxidant enzymes were not substantially activated. This effect is likely due to the experimental design, which involved a pre-treatment with a low dose of LPS (300 µg) prior to administering the full dose (600 µg). This phenomenon, known as preconditioning, occurs when an initial low-dose immune stimulation results in a mitigated response to a subsequent, higher-dose (up to lethal) stimuli. Our findings suggest that preconditioning warrants further investigation as a prospective way for mitigating inflammation-associated oxidative stress. Furthermore, our study defines a panel of inflammatory biomarkers for use in aquaculture health management and for screening the anti-inflammatory and immunostimulant activities of drugs and feed supplements. Consequently, these results could have a positive economic impact on the aquaculture industry.

## 1. Introduction

The farming of rainbow trout, *Oncorhynchus mykiss* (Walbaum, 1792), is one of the fastest-growing and continually expanding sectors of global aquaculture [1]. However, the industry’s growth is constrained by significant economic losses due to the species’ high susceptibility to infectious diseases. These infections and the associated inflammatory responses impair fish health and survival rates and can degrade product quality. Antibiotics have traditionally been used to prevent and control the spread of infectious diseases in fish stock. However, this practice has severe consequences, including the development of antibiotic resistance and the persistence of drug residues in fish tissues and the environment [2]. To prevent antimicrobial overuse, eco-friendly alternatives for disease control and welfare management are now being explored within the frameworks of One Health and sustainable aquaculture [3,4,5]. To determine the optimal timing for preventive and therapeutic interventions, there is a need to develop reliable tools based on selected reference genes and early-warning biomarkers of disease-associated inflammation.

Although the immune system of fish is physiologically similar to those of higher vertebrates, fish as poikilothermic and free-living animals are more tightly dependent on their environment. Consequently, their survival relies heavily on nonspecific immunity, which enables them to cope with multiple stressors [6,7]. Inflammation is a defense mechanism of the innate immune system, activated by foreign agents typical of pathogenic microorganisms (pathogen-associated molecular patterns, or PAMPs), trauma (damage-associated molecular patterns, or DAMPs), or other stimuli. These stimuli are recognized by selective receptor proteins in a largely nonspecific manner. Circulating acute-phase proteins, such as C-reactive protein (CRP), also recognize PAMPs (e.g., bacterial wall components, lipopolysaccharide, peptidoglycan, bacterial DNA, viral RNA, toxins) and DAMPs (e.g., particles of damaged tissues). Upon binding a foreign antigen, CRP synthesis in hepatocytes increases rapidly (within 4–6 h) and can even occur as an avalanche-like rise, at rates 10 to 100 times faster [8,9]. Under inflammatory conditions, CRP recruits effectors for antigen destruction by activating the complement system via the classical pathway, accelerating the phagocytosis, and regulating the functions of T-lymphocytes and platelets [9]. Furthermore, inflammation-activated macrophages residing in tissues and organs also produce CRP and other inflammatory mediators, such as nitric oxide (NO), prostaglandin E2, and cytokines, including interleukin 1β (IL-1β), interleukin 6 (IL-6), and tumor necrosis factor α (TNF-α) [10,11], to defend against invading pathogens. The concentration of specific molecules like CRP, cytokines, and chemokines in the bloodstream, along with their gene expression levels and the serum’s integrative capacity to eliminate microorganisms (known as serum bactericidal activity [12]), are well-established hallmarks of inflammatory processes and the host’s response to infection. Additionally, the antimicrobial activity of blood serum is reflected in clinical blood analysis indicators. Under inflammatory conditions, the composition of white blood cells undergoes quantitative changes (an inflammatory leukogram), which characterizes the systemic response to invading pathogens. The first cellular responders among leukocytes are heterophils and neutrophils. In addition to a change in their count, an inflammatory state is marked by an increase in immature granulocyte precursors, a phenomenon known as a ‘left shift’. A left shift is a clinically relevant finding in both mammalian and non-mammalian species [13]. Neutrophils are considered functionally equivalent across taxa and serve as the first line of defense against microbial (especially bacterial) infections due to their phagocytic and microbicidal activities [13,14].

In fish, visceral organs such as the liver, spleen, and kidney, are involved in the stress response, and their relative sizes—reflected by indices like the hepatosomatic index (HSI) and splenosomatic index (SSI)—can indicate exposure to environmental stressors or pathogens [15,16]. HSI correlates with metabolic and detoxifying enzyme activities in the liver; reduced hepatocyte size and alterations in nuclear morphology (in size or shape) both indicate heightened metabolic and detoxification demands [17,18]. The spleen, a key immunocompetent organ in fish, participates in immune responses and performs hematopoietic functions, primarily producing red blood cells and degrading them during senescence [19]. A decrease in the splenosomatic index (SSI) has been observed in fish exposed to unfavorable environmental factors; furthermore, the spleen typically shows a more pronounced reduction in size in response to starvation than other internal organs [20]. Inflammatory states, which involve accelerated protein synthesis and the release of aggressive mediators by immune cells and hepatocytes, lead to a surplus generation of free radicals. This necessitates the production of molecular and enzymatic antioxidants to counteract the resulting oxidative stress. Given the central role of visceral organs in regulating the immune response in fish, their relative size indices (HSI and SSI), along with antioxidant levels and enzyme activities, are considered useful markers of inflammation.

To study inflammation in fish, a well-defined model that reproduces the biochemical and molecular hallmarks of the inflammatory process must be established. One suitable model involves the systemic administration of bacterial lipopolysaccharide (LPS), a surface antigen of Gram-negative bacteria [21]. In mammals and human cell lines, LPS exhibits potent immunostimulatory activity, inducing the production of various pro-inflammatory mediators—including cytokines, chemokines and growth factors—in macrophages, fibroblasts, and monocytes to control infection [22,23,24]. Despite its experimental utility, reproducing the LPS model in fish presents challenges, particularly in determining the appropriate dosage and exposure time. Fish exhibit a higher tolerance to LPS than mammals; even in vivo exposure to high concentrations typically do not cause endotoxin-mediated mortality [25]. This phenomenon is likely due to fish-specific traits in the receptor-mediated recognition of LPS [26]. Furthermore, the conserved pathways of inflammation, LPS tolerance limits, and the set of relevant inflammatory biomarkers in fish and other non-mammalian species can differ from those in mammals [14,27]. For instance, while LPS molecular patterns are known to activate Toll-like receptor 4 (TLR4) on mammalian macrophages, most bony fishes lack a functional TLR4 ortholog, which may explain their high resistance to LPS endotoxicity [28]. The effective dosage of LPS and optimal exposure time have varied across previous studies [29,30,31] and should be determined through preliminary investigation. Nevertheless, research in trout has demonstrated that LPS exposure successively reproduces key inflammatory hallmarks, including the production of immune factors like cytokines and chemokines, the induction of oxidative stress, and the activation of the antioxidant system [25]. Therefore, the LPS model remains a valuable tool for modeling inflammatory processes in both mammals and fish.

This study utilized established markers of nonspecific innate immunity, including molecular and hematological parameters, to validate an LPS-induced inflammation model and identify representative biomarkers in rainbow trout. The validated model and the resulting panel of fish-specific inflammatory markers can be applied in aquaculture management for routine health monitoring and disease prevention. Furthermore, they provide a tool for experimental research on inflammatory physiology and pathology in fish, as well as for screening the anti-inflammatory and immunostimulatory properties of pharmaceuticals and feed supplements.

## 2. Materials and Methods

### 2.1. Experimental Fish and Rearing Conditions

All animal care and experimental procedures were approved by the PetrSU Institutional Animal Care and Use Committee (Protocol No. 1, 2 June 2025). This study was conducted in strict accordance with the ethical guidelines established by the Institutional Animal Ethics Oversight Committee of PetrSU.

A total of 80 female rainbow trout (average initial body weight: 40.3 ± 5.5 g) were obtained from the Ltd. N.V. Fedorenko trout farm (Berezovka, Kondopozhsky district, Karelia, Russia), where they were reared in a recirculating aquaculture system. The fish were transported in four 45 L barrels with continuous aeration (AIR 550 R plus four-channel air compressor, Sera, Heinsberg, Germany) to the Aquarial Base of Petrozavodsk State University. They were then acclimated to the aquarium water via gradual dilution over a six-hour period. Following acclimation, the fish were randomly distributed into four aquarium units, with 20 fish per unit. Fish mortality was checked daily; early mortality during the 10-day acclimation period was 10% (8 individuals), resulting in a final stocking density of 18 fish per aquarium. Each aquarium unit consisted of a 280 L glass tank equipped with an Atman 1200 external filter (Atman, Ningbo, China), a Hailea HC 250A external chiller (Hailea, Jiangmen, China), a Hose HK internal filter with aeration (flow rate 1800 L per h, Hose, Ningbo, China), an additional Tetra APS-400 two-channel air compressor (Tetra GmbH, Melle, Germany), and a flow-through water exchange system with a flow rate of 11 L per h.

Water quality in each aquarium was monitored daily and maintained at the following parameters: temperature, 13 °C (consistent with the source farm’s recirculating aquaculture system), measured with a ST-4 thermometer (Smartron, Yiwu, China); pH, 6.9–7.2, measured with a HI 2211-02 pH meter (Hanna, Instruments, Villingen-Schwenningen, Germany); and dissolved oxygen, >7.6 mg/L, measured with an MW 600 Pro oximeter (Milwaukee Electronics, Milwaukee, USA). Concentrations of nitrogenous compounds were maintained as follows: ammonia nitrogen < 0.018 mg/L, nitrite < 0.1 mg/L, and nitrate < 10 mg/L, assessed using NILPA-test kits (Khimfarmpribor LLC, Kursk, Russia). Major anion concentrations, determined by photometric assays, were maintained as follows: chloride < 6.7 mg/L, sulfate < 20 mg/L, and phosphate < 0.66 mg/L. The concentrations of 39 trace elements were quantified using inductively coupled plasma mass spectrometry (ICP-MS, Thermo Fisher Scientific, Waltham, MA, USA) and optical emission spectroscopy (ICP-OES, Agilent Technologies, Sante Clara, CA, USA) on the 1st and 30th days of the trial. All elements remained within permissible limits for aquaculture water quality, with no significant fluctuations observed during the experiment. A 14 h light/10 h dark photoperiod was maintained using self-adjustable lamps (150 Lm, China).

Following the four-week acclimation period, the fish were fed twice daily (10:00 and 19:00) with a commercial trout feed (AquaRex, Tver Russia; 2.5 mm granule size), identical to the diet used at the source farm. The daily ration was adjusted weekly based on fish weight to ensure complete consumption within 10 min. A feed coefficient of 2.3 was applied, following the manufacturer’s recommendations for the given water temperature. Fish were fasted for 24 h prior to the experimental treatments and until the end of the experiment.

### 2.2. Experimental Design and Sampling

**Figure 1:**
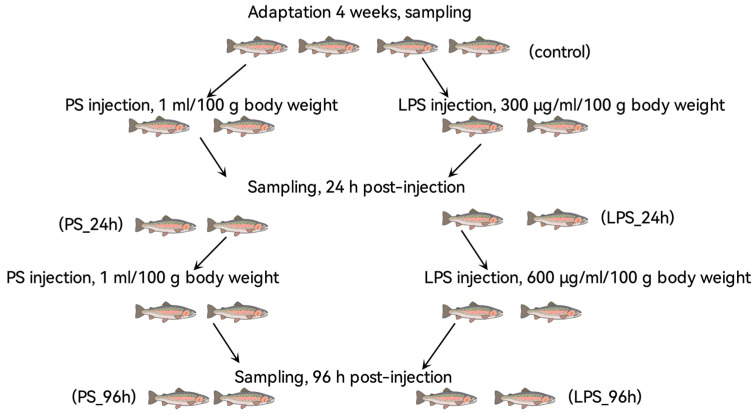
Experimental design outlining treatment administration and sampling time points. LPS: lipopolysaccharide; PS: physiological saline; 24 h and 96 h: time before sampling.

At the end of the acclimation period, 60 healthy trout (average weight: 117.81 ± 15.50 g; average length: 21.20 ± 1.43 cm) from the four tanks were randomly divided into two groups (*n* = 12 per group, with two replicate tanks per group). Throughout the experiment, basic water parameters (temperature, pH, dissolved oxygen, and nitrogenous compounds) were maintained as described in Section 2.1. One group (LPS) received an initial intraperitoneal injection of 1 mL LPS solution (300 µg per 100 g body weight), while another group (PS) received 1 mL of sterile physiological saline (per 100 g body weight). Seventy-two hours after this preconditioning treatment, the LPS group received a second injection of 1 mL LPS solution (600 µg per 100 g body weight), and the PS group received a second injection of 1 mL saline (Figure 1). Lyophilized LPS from *Escherichia coli* serotype O55:B5 (Servicebio, Wuhan, China) was used; the required dosage (300 µg or 600 µg) was dissolved in 1 mL physiological saline for administration.

To calculate the individual injection volume, fish were anesthetized in a clove oil emulsion (0.1 mL per L) until immobilized and then weighted. The solutions were administered intraperitoneally via a puncture anterior to the pelvic fin using a 2 mL syringe with a 23G needle, with care taken to avoid internal organ damage. The experiment included six biological replicates per treatment and three technical replicates per group for subsequent analyses.

Sampling was performed at three time points: pre-treatment (control; three intact fish per tank, *n* = 12 total), 24 h post-treatment (PS_24h and LPS_24h; *n* = 6 per tank from two replicate tanks, *n* = 12 per group), and 96 h post-treatment (PS_96h and LPS_96h; *n* = 6 per tank from two replicate tanks, *n* = 12 per group), as outlined in Figure 1.

For sampling, fish were euthanized by immersion in a clove oil emulsion (0.2 mL per L in a 10 L volume) until complete immobilization was achieved. Body weight and length were measured to calculate morphometric indices. Blood was collected from the caudal vein using a 2 mL vacutainer tube containing a clotting activator. The sample was centrifuged at 4000 rpm for 10 min to separate the serum. A 200 µL aliquot of serum was stored at −80 °C for C-reactive protein (CRP) analysis. The remaining serum was kept at +4 °C in a microcentrifuge tube and used for the serum bactericidal activity (SBA) assay within 24 h. Additionally, two separate 2 µL blood samples were collected from the caudal vein using an EDTA-coated vacutainer tube for the preparation of blood smears for microscopic examination. Following blood collection, fish were dissected, and the liver and spleen were excised and weighted to calculate the hepatosomatic index (HSI) and splenosomatic index (SSI).

Liver samples were stored at −80 °C for subsequent antioxidant enzyme assays. Spleen samples were immediately preserved in RNA stabilization buffer (Extract RNA, Evrogen, Moscow, Russia) for future cytokine expression analysis.

### 2.3. Morphometric and Organosomatic Indices

Based on individual measurements, Fulton’s condition factor and organosomatic indices were calculated using the following formulae:

Fulton’s condition factor (K) = [body weight (g)/(body length (cm))^3^] × 100

Hepatosomatic index (HSI, %) = [liver weight (g)/body weight (g)] × 100

Splenosomatic index (SSI, %) = [spleen weight (g)/body weight (g)] × 100

### 2.4. Dose-Finding Study

A preliminary dose-finding study was conducted to determine the optimal LPS concentration for inducing a clinically significant, non-lethal inflammatory response in rainbow trout. Fish of similar size (average weight: 110.11 ± 18.85 g) were divided into four groups (*n* = 6 per group). Three groups received intraperitoneal injection of LPS at 50, 125, or 500 µg per 100 g body weight (dissolved in 1 mL physiological saline), while a control group received saline only, at 1 mL per 100 g body weight. Inflammatory responses were assessed at 24 and 72 h post-injection. Assessment included (1) clinical examination for morphological changes (e.g., reddened abdomen, petechial hemorrhages on the skin and fins); (2) post-mortem pathoanatomic examination for signs of intestinal inflammation (e.g., edematous internal organs, enteritis, hemorrhages in the abdominal cavity and visceral fat), and (3) hematological analysis via microscopic examination of blood smears for changes in white blood cell composition (e.g., leukocytosis, neutrophilia, etc.). Based on these observations, a single administration of 300–400 µg LPS per 100 g body weight was determined to be optimal for inducing a clear inflammatory response in trout.

### 2.5. Analysis of il1ß and il8 Gene Expression in Spleen

Total leukocyte RNA was extracted from 50 mg of spleen tissue using Extract RNA reagent (Evrogen, Moscow, Russia) according to the manufacturer’s instructions, with minor modifications. Briefly, homogenized samples were mixed with 200 μL chloroform and centrifuged at 12,000 rpm for 5 min at +4 °C (Allegra 64R centrifuge, Beckman Coulter, Brea, CA, USA). The aqueous phase was transferred to a new tube, mixed with an equal volume of isopropanol, and centrifuged again to pellet RNA. The pellet was washed twice with 80% ethanol, air-dried for 5 min, and resuspended in 100 μL of nuclease-free water. Residual genomic DNA was removed with DNase I (1 U, Sibenzyme, Novosibirsk, Russia) at 37 °C for 30 min. RNA quantity and integrity were assessed via agarose gel electrophoresis. Complementary DNA (cDNA) was synthesized from 1 μg of total RNA using the MMLV RT kit (Evrogen, Moscow, Russia) according to the recommendation of the manufacturer. Quantitative real-time PCR (qRT-PCR) was performed in a CFX96 Touch system (Bio-Rad, Irvine, CA, USA) using Screen-Mix SYBRGreen reagents (Evrogen, Moscow, Russia). The thermal cycling protocol consisted of an initial denaturation at 95 °C for 3 min, followed by 40 cycles of 95 °C for 30 s, 58 °C for 30 s, and elongation at 72 °C for 30 s. Amplification efficiency, determined from the standard curve of serial cDNA dilutions, was 98–100% for all genes. The relative transcript levels of *il1ß* and *il8* were calculated using the ΔΔCt method [32], with normalization to the geometric mean of two reference genes, elongation factor-1 alpha (*ef1α*) and ß-actin (*actb*). Each sample was run in at least three technical replicates. Primer sequences were borrowed from the available sources and listed in Table 1.

### 2.6. Determination of Serum Bactericidal Activity (SBA)

The bactericidal activity of trout serum against the *E. coli* reference strain O55:B5 was assessed using a turbidimetric assay [34] as described below.

First, the bacterial growth kinetics were characterized to determine the optimal culture stabilization time. A lyophilized *E. coli* culture was inoculated into three sterile flasks containing 200 mL of meat-peptone broth (MPB) using a sterile microbiological loop. The flasks were incubated at 37 °C (TS-1/20 SPU thermostat, SKTB SPU, Smolensk, Russia) alongside a blank MPB control (volume sufficient for 5–6 measurements) in a separate flask. The optical density at 605 nm of culture aliquots (in three replicates from each flask) was measured every 24 h against the blank using a microplate reader (Allsheng, Guangdong, China) until the culture reached the stationary phase (stabilization time).

For SBA assay, a fresh culture was prepared by inoculating 200 mL of MPB and incubating it for the predetermined stabilization time. A 100 mL subculture was then inoculated from this and incubated at 37 °C for 24 h. The assay was performed in a 24-well culture plate. Each well contained 100 µL of the 24 h bacterial culture, 2.5 mL of MPB, and 0.5 mL of trout serum. Serum from 12 fish per group (*n* = 6 from each of the two replicate tanks) was analyzed, with each serum sample tested in three technical replicates. A positive control well, containing 100 µL of bacterial culture and 3 mL of MPB without serum, was included to represent uninhibited bacterial growth. The optical density at 605 nm of both control (Econtrol) and experimental (Eexp) wells was measured immediately and after 3 h of incubation at 37 °C. The serum bactericidal activity (SBA) was calculated using the following formula:

SBA = (ΔEcontrol − ΔEexp)/ΔEcontrol.

### 2.7. Serum and Hepatic Biochemical Analyses

Serum was used for all analyses to avoid potential interference from fibrinogen present in plasma. The concentration of C-reactive protein (CRP) in the serum was quantified using a commercial Fish High-Sensitivity C-reactive Protein ELISA kit (BlueGene, Shanghai, China), following the manufacturer’s protocol for a solid-phase direct competitive enzyme immunoassay. The optical density of the reaction products was measured at 450 nm using a CLARIOStar microplate reader (BMG Labtech, Ortenberg, Germany). Each sample was analyzed in at least two technical replicates, and CRP concentrations were determined from a standard calibration curve.

For hepatic analyses, liver tissue was homogenized for 2 min in ice-cold 50 mM Tris-buffer saline (pH 7.5, 1:4 *w*/*v*) using a Tissue Lyser LT (QIAGEN N.V., Hilden, Germany). The homogenate was centrifuged at 45,000 rpm for 60 min at 4 °C (Allegra 64R centrifuge, Beckman Coulter, Brea, USA), and the resulting supernatant was collected. Soluble protein concentration was determined by measuring the specific absorption of the peptide bond at 220–230 nm [35,36], superoxide dismutase (SOD) activity was assessed by its inhibition of adrenaline auto-oxidation at 480 nm [37], catalase (CAT) activity was measured by tracking the consumption of H_2_O_2_ at 240 nm [38], and glutathione-S-transferase (GST) activity was determined by measuring the conjugation rate of reduced glutathione with 1-chloro-2,4-dinitrobenzene (CDNB) at 340 nm [39].

### 2.8. Blood Smear Microscopy

Duplicate thin blood smears were prepared for each sample by expressing 2 µL of fresh blood onto a clean glass slide and spreading it using a spreader slide. The smears were air-dried and fixed with methanol-based May-Grünwald stain fixative (Minimed, Bryansk, Russia) for 3 min. After fixation, slides were rinsed with running water, air-dried, and then stained for 20 min with a Romanowsky Azure-Eosin dye solution (prepared in a 5:4:1 ratio of dye, distilled water, and phosphate buffer, pH 6.4–6.8). Following a final rinse and air-drying, the prepared smears were examined under a light microscope (Olympus CX-41, Tokyo, Japan) under oil immersion at 1000× total magnification (100× objective lens and 10× ocular lens). For each smear, leukocyte differential counts were performed by assessing at least 30 fields of view.

### 2.9. Statistical Analysis

All analyses and visualizations were performed using R (R Core Team, Vienna, Austria) within the RStudio ver. 2025.09.1 integrated development environment (Posit team, Boston, MA, USA). The normality of data distribution was assessed with the Shapiro–Wilk test. A comparative analysis of the indicators was conducted using the Kruskal–Wallis test and Dunn’s post hoc with a correction for multiple Bonferroni comparisons (“rstatix” package). Statistical significance was defined as a *p*-value ≤ 0.05.

## 3. Results

### 3.1. Morphometric and Organosomatic Indices and Welfare of Fish

The biometric indices of the experimental fish are presented in Table 2. The average weight was 40.3 ± 5.5 g. Mortality was 10% (eight individuals) during the initial ten days of the acclimation period, with no mortality occurring after the experimental injections. Following the four-week acclimation period, the average fish weight increased 3.5-fold to 120.3 ± 15.4 g, and the average length (AB) was 21.3 ± 0.9 cm. At the start of the experiment, no significant differences in weight or Fulton’s condition factor (K) were detected between the groups. A significant decrease in HSI was observed 24 and 96 h after the PS injection, as well as 96 h after LPS injection. In contrast, K and SSI showed no significant differences between any of the treatment groups at any time point.

**Figure 2:**
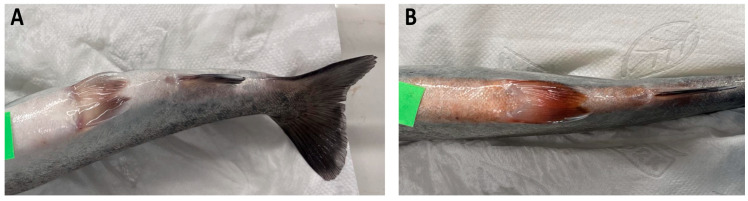
Rainbow trout 24 h post-injection with (**A**) physiological saline (PS) or (**B**) lipopolysaccharide (LPS). Representative manifestations of LPS-induced inflammation included abdominal hemorrhages and erythema.

Visible signs of inflammation, including hemorrhages and erythema (“red marks”) on the abdonimal surface of body and fins developed several hours after the intraperitoneal injection of LPS but not in the saline-injected controls (Figure 2).

### 3.2. Interleukin Gene Expression in the Spleen

**Figure 3:**
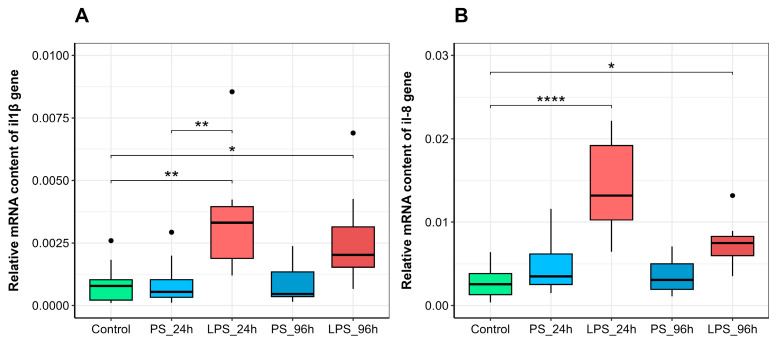
Relative gene expression of (**A**) *il1β* and (**B**) *il8* in the spleen of rainbow trout injected with physiological saline (PS) or lipopolysaccharide (LPS), sampled 24 h or 96 h post-injection. Significance levels: * *p* ≤ 0.05, ** *p* ≤ 0.01, **** *p* ≤0.0001.

The expression of genes encoding the pro-inflammatory cytokines interleukin-1β and interleukin-8 was significantly upregulated in the spleen following LPS administration compared to saline-injected controls (Figure 3). This confirms a specific inflammatory gene response to LPS in trout.

### 3.3. Serum Bactericidal Activity (SBA)

**Figure 4:**
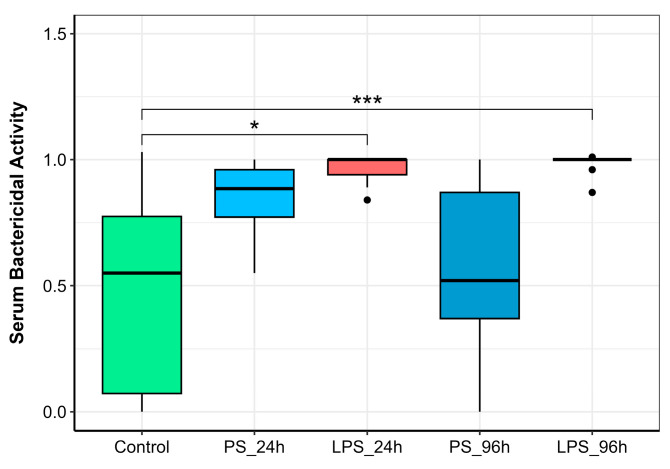
Serum bactericidal activity (SBA) in rainbow trout injected with physiological saline (PS) or lipopolysaccharide (LPS) and sampled 24 h or 96 h post-injection. Significance levels: * *p* ≤ 0.05, *** *p* ≤ 0.001.

Intraperitoneal administration of 300 µg LPS resulted in a significant 1.6-fold increase in SBA within 24 h (LPS_24h group) compared to the control (Figure 4). Following the subsequent 600 µg LPS injection, SBA remained significantly elevated at 96 h (LPS_96h). In contrast, administration of physiological saline (PS) had no significant effect on SBA at either 24 or 96 h post-injection.

### 3.4. Serum C-Reactive Protein (CRP) Concentration

**Figure 5:**
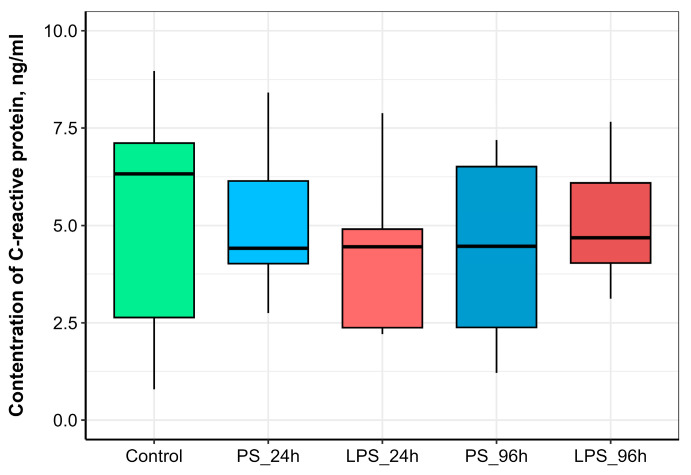
Serum CRP concentration in rainbow trout injected with physiological saline (PS) or lipopolysaccharide (LPS) and sampled at 24 h or 96 h post-injection.

Serum CRP levels showed no significant changes at 24 h after a single injection or at 96 h after the double injection in either the LPS or PS treatment groups compared to the control (Figure 5).

### 3.5. Hepatic Antioxidant Enzyme Activities

**Figure 6:**
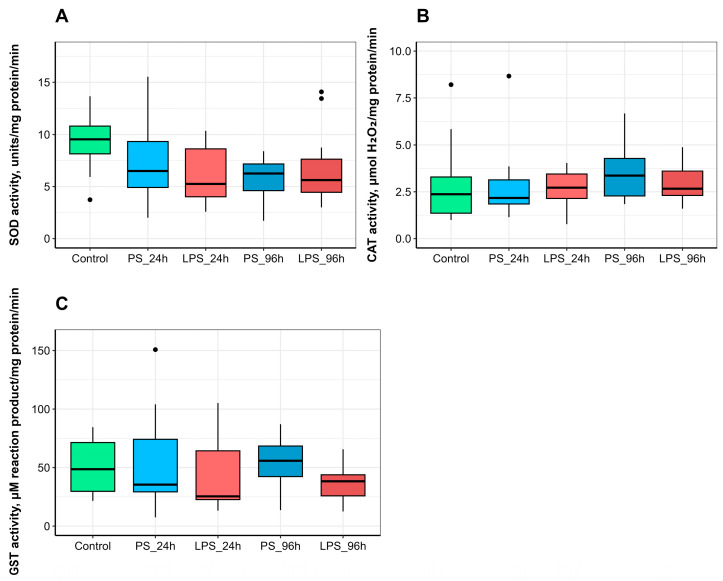
Hepatic activities of antioxidant enzymes: (**A**) superoxide dismutase (SOD), (**B**) catalase (CAT), (**C**) glutathione S-transferase (GST), in rainbow trout injected with physiological saline (PS) or lipopolysaccharide (LPS) and sampled at 24 h or 96 h post-injection.

No significant differences were observed in the hepatic activities of superoxide dismutase (SOD), catalase (CAT), or glutathione S-transferase (GST) between the control group and any treatment group (PS or LPS) at either 24 or 96 h post-injection (Figure 6).

### 3.6. Peripheral Blood Leukocyte Profiles

**Figure 7:**
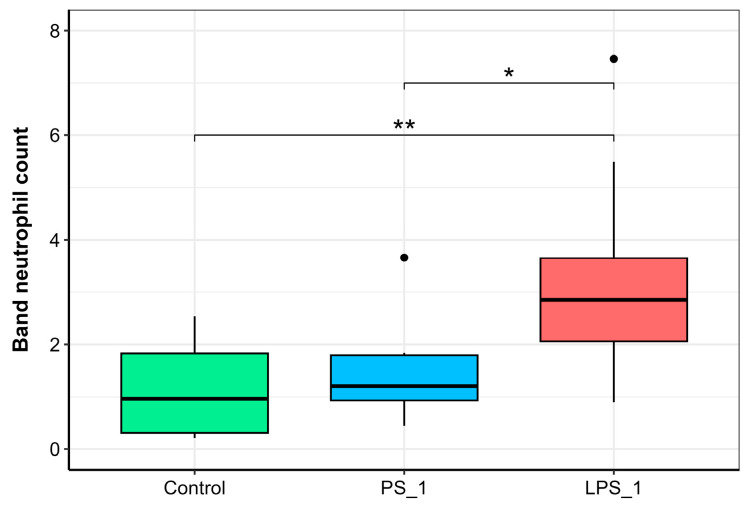
Band neutrophil counts in the peripheral blood of rainbow trout injected with physiological saline (PS) or lipopolysaccharide (LPS) 24 h post-injection. Significance levels: * *p* ≤ 0.05, ** *p* ≤ 0.01.

Microscopic analysis of peripheral blood smears identified five distinct cell types: erythrocytes, neutrophils (including precursor, band, and segmented forms), lymphocytes, monocytes, and thrombocytes. Lymphocytes were the predominant leukocyte (>50% of total leukocytes), followed by neutrophils (15–20%) as the main granulocyte, and monocytes (<5%). Other granulocytes, such as basophils and eosinophils, were not observed. Among all hematological variables, the count of immature (band) neutrophils exhibited a specific, two-fold increase (*p* ≤ 0.01) at 24 h after LPS injection (Figure 7). No other leukocyte types showed a significant response to LPS. In contrast, fish injected with saline showed no significant quantitative or qualitative alterations in their leukocyte profiles.

## 4. Discussion

As poikilotherms, fish are more tightly dependent on the environment and are in constant contact with potential pathogens. Consequently, their survival depends heavily on a robust nonspecific immune system that can be rapidly activated to counter diverse environmental stressors [6,7]. This also means fish can be highly vulnerable to secondary stressors, such as the physiological burden of inflammation following injury or infection [40]. The development of effective diagnostic and therapeutic approaches for inflammation in fish must therefore be grounded in a panel of verified inflammatory markers, defined using a validated experimental model. The LPS-induced inflammation model is widely used due to its cost-effectiveness, reproducibility, and representation of key inflammatory hallmarks. While inflammatory biomarkers in this model are well-characterized in mammals such as mice [24], the evidence in fish remains limited. It is established that LPS induces an innate immune response in fish, as in other vertebrates, activating numerous immune-related genes in immune cells and tissues, including in trout [25,41,42]. In this study, we utilized a core set of established inflammatory markers to assess the suitability of the LPS model for investigating inflammatory physiology in rainbow trout, even while acknowledging that the specific hallmarks of inflammation may differ in cold-blooded vertebrates [27].

In this study, rainbow trout received two consecutive intraperitoneal injections of LPS at 300 µg and 600 µg per 100 g body weight. This design was based on the established concept of preconditioning, where a sublethal dose of a damaging agent can induce a state of cross-tolerance, enhancing the organism’s ability to withstand a subsequent, more severe insult [43,44,45]. For instance, in neuroinflammation models, sublethal LPS preconditioning has been shown to confer neuroprotective and anti-apoptotic effects by modulating metabolic activity, activating specific signaling pathways, and stimulating the immune system, thereby mitigating later cytotoxic damage [46]. This strategy is a promising avenue for uncovering endogenous defense and repair mechanisms that could be therapeutically harnessed to counteract pro-inflammatory effects.

Determining an appropriate LPS concentration to induce a representative yet sublethal inflammatory response in rainbow trout is challenging, as the existing literature provides limited guidance, with recommendations ranging from 600 to 900 μg per 100 g body weight [21,47]. To address this, we conducted a preliminary dose-finding study. This investigation confirmed that a dosage of 500 μg LPS successfully elicited key inflammatory hallmarks, including leukocytosis and intestinal edema. Based on these results, we selected an initial, lower dose of 300 µg per 100 g body weight to serve as the preconditioning stimulus. The subsequent, higher dose of 600 µg was intended to act as a significant inflammatory challenge that would be better tolerated by preconditioned fish compared to naive individuals.

In fish, visceral organs such as the liver, spleen, and kidney are integral to innate immunity [48]. Consequently, indices like the hepatosomatic index (HSI) and splenosomatic index (SSI) reflect the metabolic and immune resources available to cope with stressors, including pathogens and environmental challenges [15,16,17,49]. The progressive decrease in HSI observed from 24 to 96 h after injection—in both LPS and saline groups (Table 2)—likely resulted from a combination of the traumatic injury caused by the injection procedure itself and the mobilization of hepatic energy reserves in fasted fish.

In our study, the significant upregulation of *il1β* and *il8* gene expression following LPS administration, but not saline, confirms the specific pro-inflammatory effect of LPS in trout (Figure 3A,B). In vertebrates, including various fish species, IL-1β and IL-8 are key mediators of acute inflammation, coordinating the systemic response to infection and tissue damage, and activating antimicrobial defenses such as lysozyme and other cytokines [50,51]. Our results demonstrate that even a low-dose LPS stimulus (300 µg) was sufficient to trigger a significant cytokine response in the spleen within 24 h.

Interestingly, the magnitude of the cytokine response was similar following both the initial (300 µg) and the subsequent, higher (600 µg) LPS injection. This suggests that the gene expression response was either dose-independent or, more likely, modulated by a preconditioning effect. While the difference in il8 expression between the single and double injection groups was not statistically significant according to our criteria (*p* > 0.05), it is notable that the second, higher-dose injection did not produce a stronger response than the first. This attenuated response to the second stimulus is consistent with a preconditioning effect, where the initial low-dose exposure induces a tolerant state, mitigating the response to subsequent challenge.

Serum bactericidal activity (SBA) represents the integrated effect of multiple humoral immune factors in the bloodstream, such as the complement system, lysozyme, antimicrobial peptides, and lectins [11,52]. Beyond infectious challenges, SBA can be enhanced by various environmental stressors, serving as a valuable indicator of an organism’s physiological and immune status [7]. In our experiment, LPS administration at both 300 µg and 600 µg significantly increased SBA compared to the control group (*p* < 0.05 and *p* < 0.001, respectively; Figure 4). The complement system, a potent effector mechanism activated by inflammatory agents like LPS, is likely a primary contributor to this observed increase in bactericidal capacity [53]; however, further studies are required to delineate the exact contributions of individual humoral components.

Acute-phase proteins (APPs), such as C-reactive protein (CRP), are produced by hepatocytes upon stimulation by pro-inflammatory cytokines released from macrophages and other cells during infection or tissue damage [54]. Teleost fish express several APPs homologous to those in mammals, and CRP is known to play a key role in the piscine immune response. For example, studies have reported a three-fold increase in CRP in rainbow trout infected with *Vibrio anguillarum* [11] and an 18-fold increase after formalin exposure [55]. In contrast, our study found that serum CRP levels were not significantly elevated by either the 300 µg or 600 µg LPS dose, nor by the physical trauma of the injection itself. This lack of response was unexpected. We hypothesize that the initial LPS dose (300 µg), while sufficient to activate other innate immune mechanisms like cytokine expression and SBA, was below the threshold required to trigger a significant systemic acute-phase response involving CRP. Furthermore, the subsequent higher dose (600 µg) was administered to preconditioned fish, a state which may have actively suppressed the CRP response as part of the cross-tolerance mechanism, thereby preventing the pronounced increase typically seen in a naive inflammatory challenge.

Oxidative stress, resulting from an imbalance between oxidation and antioxidant capacity, can lead to inflammatory neutrophil infiltration, increased protease activity, the accumulation of oxidized macromolecules, and subsequent tissue damage [56]. We hypothesized that both LPS administration and the intraperitoneal injection procedure itself could act as stressors, potentially eliciting a response from the antioxidant system (AOS). Key enzymes in the primary antioxidant defense include superoxide dismutase (SOD) and catalase (CAT), which directly neutralize reactive oxygen species [57]. Glutathione S-transferase (GST) also contributes to antioxidative capacity by facilitating the conjugation of glutathione (GSH) with foreign substances during Phase II biotransformation. Although antioxidant enzyme activities are widely used as biomarkers of oxidative stress, findings across studies are often inconsistent [58]. For instance, LPS exposure has been reported to induce oxidative stress in some fish species, as evidenced by decreased CAT and SOD activities and increased lipid peroxidation in the gut and hepatopancreas [31,59]. Conversely, other research indicates that LPS does not induce oxidative stress or alter antioxidant enzyme expression in fish muscle cells [60]. Our results align with the latter perspective. We observed no significant effect of either the 300 µg or 600 µg LPS dose on the activities of SOD, CAT, or GST in the trout liver at 24 or 96 h post-injection. The lack of a response to the physical trauma of the injection itself further supports the conclusion that the treatments did not induce significant oxidative stress in the liver of the studied trout.

Blood serum and leukocytes constitute the primary defense against invading pathogens through mechanisms such as phagocytosis and cell lysis [61]. In fish, leukocytes play a predominant role in combating infection, making their differential counts a valuable diagnostic tool for assessing health status [62], though these counts are influenced by factors like stress, age, and rearing conditions [63]. Lymphocytes are the most abundant leukocyte in the peripheral blood of fish, constituting >50% of total leukocytes, which is a markedly higher proportion than in mammals [64,65]. In our study of *O. mykiss*, the remaining leukocyte population consisted of neutrophils (15–20%) as the primary granulocyte and monocytes (<5%); basophils and eosinophils were not observed. The leukocyte profile is a sensitive indicator of the physiological state and reflects the organism’s response to stressors and pathogens [66].

In our study, a significant two-fold increase in band neutrophils was observed 24 h after LPS administration, indicating a pronounced inflammatory response characterized by a “left shift” (Figure 7). This increase in immature granulocytes is a classic hematological marker of acute inflammation, representing an adaptive mechanism to mobilize the innate immune system against threat [13]. Our findings are consistent with those of Havixbeck et al. [64], who reported neutrophilia in *Carassius auratus* following zymosan injection, underscoring the conserved, effector role of neutrophils as a first line of defense in fish. The primary function of neutrophils is to rapidly migrate to sites of inflammation and eliminate pathogens. Our results confirm their activation and recruitment into the circulation following an immune challenge. The modern understanding of neutrophil function has expanded beyond phagocytosis to include the production of reactive oxygen and nitrogen species, degranulation of antimicrobial enzymes, and the release of neutrophil extracellular traps (NETosis) [67]. The elevated neutrophil count, coupled with the specific increase in immature forms, serves as a clinical hallmark of acute inflammation in trout. Consequently, the detection of a left shift through blood smear microscopy provides high diagnostic value and can be recommended as a rapid, effective tool for assessing fish welfare and identifying inflammatory states in aquaculture settings.

## 5. Conclusions

In this study, we validated an LPS-induced inflammation model in rainbow trout and identified a panel of specific biomarkers suitable for monitoring inflammatory states. We experimentally established that a dose of 300–500 µg LPS per 100 g body weight elicits a clinically significant inflammatory and innate immune response. Interpretation of the LPS-induced effects observed in this study must account for influencing variables such as water temperature, feeding regime, LPS dose, and the age and size of the fish. Our results demonstrate that key hallmarks of this response include the upregulation of pro-inflammatory cytokines (*il1β* and *il8*), a neutrophilia characterized by a distinct left shift, and a significant increase in serum bactericidal activity. Conversely, some classic mammalian inflammatory markers, such as a systemic acute-phase protein (CRP) response and hepatic antioxidant enzyme activation, proved to have low diagnostic value for the low-grade inflammation reproduced in our trout model. It remains to be determined whether this reflects a physiological trait of rainbow trout or is a consequence of our specific experimental conditions, including water temperature, LPS dosages, and the preconditioning protocol. A key finding was the observation of a preconditioning effect. The initial low-dose LPS treatment mitigated the inflammatory response to the subsequent higher dose, rather than producing an additive effect. The early and detectable responses in interleukin expression, SBA, and granulocyte profiles suggest these parameters have high potential as biomarkers for the routine veterinary monitoring of fish health. Furthermore, this validated model and biomarker panel provide a robust methodological foundation for screening the anti-inflammatory and immunostimulant properties of candidate pharmaceuticals and feed supplements in aquaculture.

## Figures and Tables

**Table 1:** Primer sequences and amplicon lengths for RT-PCR.

Gene	Sequence 5′ → 3′, F–Forward, R–Reverse	NCBI Accession Number	Amplicon Length, n.p.	Reference
*actb*	F:CCGGCCGCGACCTCACAGACTAC R:CGGCCGTGGTGGTGAAGCTGTAAC	NM_001124235	73	[11]
*ef1α*	F: GGTGGTGTGGGTGAGTTTGAG R: AACCGCTTCTGGCTGTAGGG	NM_001124339	159	[33]
*il1ß*	F: ACCGAGTTCAAGGACAAGGA R: CATTCATCAGGACCCAGCAC	NM_001124347	181	[11]
*il8*	F: TGTCGTTGTGCTCCTGG R: CCTGACCGCTCTTGCTC	NM_001124362.1	197	[11]

**Table 2:** Morphometric and organosomatic indices of rainbow trout, *O. mykiss*.

Treatment/Index	Control, Pre-Treatment	PS_24h	LPS_24h	PS_96h	LPS_96h
K	1.25 ± 0.04	1.15 ± 0.06	1.12 ± 0.04	1.09 ± 0.02	1.09 ± 0.07
HSI, %	1.17 ± 0.09	0.99^a^ ± 0.14	1.17 ± 0.07	0.82 ± 0.06 ^a,b^	0.93 ± 0.04 ^a,b,c^
SSI, %	0.09 ± 0.04	0.08 ± 0.05	0.11 ± 0.02	0.05 ± 0.02	0.08 ± 0.04

The data are presented as median ± 1/2 IQR. a—significant difference from the control group (*p* ≤ 0.05); b—significant difference between PS and LPS treatments at the same time point (*p* ≤ 0.05); c—significant difference between the 24 h and 96 h post-injection groups (*p* ≤ 0.05).

## Data Availability

The raw data supporting the conclusions of this article will be made available by the authors on request.

## Abbreviations

The following abbreviations are used in this manuscript:

CAT	catalase
CRP	C-reactive protein
GST	glutathione-G-transferase
HSI	hepatosomatic index
il1ß	interleukin 1β
il8	interleukin 8
LPS	lipopolysaccharide
PS	physiological saline
SBA	serum bactericidal activity
SOD	superoxide dismutase
SSI	splenosomatic index
